# Rapid Epidemiological Data Collection on Social Media for COVID-19: Comparative Study Between Online Surveys and Conventional Cohorts

**DOI:** 10.2196/80311

**Published:** 2026-04-27

**Authors:** Maged Mortaga, Hendrik Nunner, Sydney Paltra, Leonard Stellbrink, Jens Friedel, Manuela Harries, Jessica Krepel, Berit Lange, MuSPAD Study Group, Viola Priesemann, André Calero Valdez

**Affiliations:** 1 Institute of Human-Centered Interactive Systems (IMIS), University of Lübeck, Ratzeburger Allee 160, Lübeck, 23562, Germany, 49 451-3101 5117; 2 Technische Universität Berlin, Berlin, Germany; 3 Max Planck Institute for Dynamics and Self-Organization, Göttingen, Germany; 4 Helmholtz Center for Infection Research, Braunschweig, Germany; 5 Institute for the Dynamics of Complex Systems, Faculty of Physics, University of Göttingen, Göttingen, Germany

**Keywords:** cross-sectional studies, pandemics, data collection, communicable diseases, social media, Twitter, Mastodon, COVID-19, Multilocal and Serial Prevalence Study of Antibodies Against Respiratory Infectious Diseases, MuSPAD, COVID-19 Snapshot Monitoring, COSMO, epidemiological models, digital health

## Abstract

**Background:**

After COVID-19 was declared a pandemic by the World Health Organization (WHO) in March 2020, global responses relied on nonpharmaceutical interventions such as physical distancing and mask mandates. These measures were guided by mathematical models built on empirical data. Although traditional methods such as surveys and observational studies provide high-quality data, they are often slow and resource-intensive. Social media polls (SMPs) offer a faster, more cost-effective alternative.

**Objective:**

This study aims to evaluate the reliability and biases of SMPs as a rapid supplementary tool for epidemiological data collection and to compare their representativeness and data quality with conventional approaches.

**Methods:**

In this cross-sectional observational study in Germany, we used SMPs to collect data on infections and demographic attributes via Twitter and Mastodon. We collected data directly on the social media platforms as well as through forwarding to an external survey via post. The time frame covered was from 2019 to 2024. Data were analyzed for infection rates, sociodemographic representativeness, and overall data quality.

**Results:**

SMPs demonstrated viability as a rapid data collection tool. Based on a sample of 6127 answers on social media and 867 responses from the external survey, the self-reported frequency of infection aligned well with conventional sources. Across all 4 studies, approximately one-third of respondents reported having never been infected, half reported having had 1 infection, and one-sixth reported having had 2 or more infections. Statistical analyses of differences between data from Twitter, Mastodon, the external survey, and conventional data showed only small effect sizes (Cohen *w*=0.105‐0.188). Spearman rank correlation demonstrated strong positive associations between infection dates in the external survey and conventional data (ρ=0.883, *P*<.001), as well as between the external survey and the Robert Koch Institute (ρ=0.640, *P*<.001). However, demographic analyses revealed biases in the external survey. By design, SMPs do not provide detailed demographic data, limiting options for subgroup analyses.

**Conclusions:**

We found SMPs to be a practical and cost-effective method for quickly gathering epidemiological insights. In particular, self-reported infection frequency can aid during periods of high availability of self-testing during epidemics. We demonstrate that, even with a nonrepresentative and biased sample, we were able to closely match infection numbers with Multilocal and Serial Prevalence Study of Antibodies Against Respiratory Infectious Diseases in Germany data and produce incidence trends comparable to those in official Robert Koch Institute data. One can argue that SMPs alone are insufficient for public health modeling, as they do not allow real-time monitoring of, for example, population infection rates based on serological data. They are also limited with regard to inherent demographic bias related to recruitment and the inability to collect individual-level covariates. However, they can complement traditional approaches by offering rapid, low-cost insights.

## Introduction

After the World Health Organization declared COVID-19 a public health emergency of international concern on January 30, 2020 [1] and as a pandemic on March 11, 2020 [2], it triggered unprecedented global responses aimed at mitigating the spread of infections and caused widespread societal, psychological, and economic impacts [3-7]. In the absence of an effective vaccine, various nonpharmaceutical intervention measures (NPIs) to contain the spread of the virus were implemented. NPIs included recommendations for physical distancing, travel restrictions, hygiene and sanitation measures (such as mask mandates), and temporary lockdowns [8-16]. Policy decisions (eg, NPIs, vaccination recommendations) were based on various sources of empirical data, such as case numbers, surveys on public behavior and attitudes, mobility changes, and social contacts [17], which were extensively supported by mathematical models to estimate the efficacy of such measures [18-24]. Such models, however, require high-quality and rapidly collected empirical data to serve as a reliable source of information for public health decisions [25-27].

There are different ways of collecting empirical data, which differ in collection speed and the quality of information. Conventional methods typically produce high-quality information while requiring a long time for data collection. For example, in Germany, seroprevalence studies like the Multilocal and Serial Prevalence Study of Antibodies Against Respiratory Infectious Diseases (MuSPAD) in Germany [28] provided valuable insights. The MuSPAD study uses established protocols to measure the prevalence of antibodies against SARS-CoV-2 in the population at different times to determine when and how many people have been exposed to the virus [29]. It was later adapted to an epidemic panel and supplemented by novel multiplex serological devices able to gather reinfection data for relevant respiratory infections. Another example is the COVID-19 Snapshot Monitoring (COSMO) study [30], which uses repeated cross-sectional surveys to continuously track public perceptions, attitudes, and behaviors regarding the COVID-19 pandemic in Germany to inform public health interventions and improve communication strategies. While these approaches provide high-quality data from representative samples, they are time-consuming and costly, limiting their ability to inform real-time public health decisions. In contrast, novel methods, such as scraping social media data, typically allow faster data collection, however, at the cost of producing lower-quality information [31-33]. Social media data can be less reliable due to several factors, including demographic disparities, selection, self-selection bias, inconsistent user activity levels, and platform bias [34-36]. Previous studies have identified these biases in social media data, particularly in the context of public health crises, such as demographic differences in posting COVID-19-related content [37] and the challenges of ensuring representativeness in Twitter (We will refer to X as Twitter throughout this paper, as that was the official name during data collection.) polling data [38]. In addition, social media was one of the main drivers of spreading misinformation during the COVID-19 pandemic [39-41], with WhatsApp, Facebook, Twitter, and YouTube among the most used platforms [42-44]. Despite these challenges, social media polls (SMPs) offer the advantage of collecting large amounts of data in real-time without labor- and cost-intensive processes. This has been shown, especially in health-related contexts [38,45-47].

In addition to direct data collection, SMPs hold the potential to serve as a rapid and cost-effective recruitment tool for more comprehensive linked online surveys. Such surveys can capture more nuanced and extensive epidemiological information. By leveraging SMPs for recruitment, researchers can therefore balance cost-efficiency and the need for high-quality data.

However, a systematic assessment of the practicability, reliability, and biases of data collected using SMPs and comparison of data quality with conventional epidemiological methods has not been undertaken, leaving significant gaps in understanding the potential of SMPs in epidemiological contexts. We therefore ask: to what extent does COVID-19 data collected through SMPs differ from conventional methods regarding representativeness and reliability?

To test the reliability of SMPs both as data sources and recruitment tools for epidemiological surveys, we hypothesize the following:

- H1: Data related to COVID-19, sourced from cost-effective SMPs and shared surveys, is less representative of the population compared to data derived from more conventional methods, such as the MuSPAD antibody study. However, we assume that the differences show only small effects.

To test the reliability of SMP data in terms of the platform used for data collection, we hypothesize the following:

- H2: The type of social media platform used for gathering data will significantly influence the selectivity of the population represented and, consequently, the relevance and reliability of the data collected.

## Methods

### Study Design and Objectives

This study employed a quantitative cross-sectional design to evaluate the reliability and biases of SMPs compared to conventional epidemiological methods for collecting health-related data. We used Twitter and Mastodon, a decentralized social media platform, to gather data on COVID-19 infections, integrating this approach with a linked online LimeSurvey questionnaire. Additionally, data from conventional sources, including the MuSPAD and COSMO studies, as well as official reports from the Robert Koch Institute (RKI) and Federal Statistical Office of Germany (FSO), were used for comparison.

Our study was preregistered on OSF in July 2023 [48]. Our third preregistered hypothesis focused on the derivation of contact network properties (H3: During the COVID-19 pandemic, individuals exhibited more pronounced social selectivity based on a homophily—the tendency to associate with similar others—due to increased awareness of their “second-order contacts” [contacts of contacts]) is tested in a second, forthcoming publication [49]. The recruitment strategy was developed afterward to increase reach and was introduced during the survey development. Bootstrapping was also introduced at a later point to address recruitment bias.

### Ethical Considerations

This study was conducted in accordance with the Declaration of Helsinki. Informed consent was obtained from all participants. It was approved by the ethics committee of the University of Lübeck (approval number 2023-413). Reporting was conducted in accordance with the STROBE (Strengthening the Reporting of Observational Studies in Epidemiology) reporting guidelines [50]. Recruiters were not compensated in any way and included study authors, as well as one person affiliated with the study institutions. Sharing the recruitment link via social media merely served as a neutral means of participant recruitment and did not provide the authors with any personal, financial, or competitive advantage influencing the study’s outcomes.

Clarification and obtaining consent to share nonpersonal data as part of a social media survey took place in terms of use of the respective social media services. Apart from the publicly accessible survey data, no other, and thus, in particular, no personal data are used.

### Recruiters and SMP Distribution

We enlisted 5 German-speaking recruiters, each with an established social media presence, to distribute the SMPs. All recruiters were informed about the study and willingly agreed to participate. The recruiters had follower counts ranging from approximately 750 to 65,000 on Twitter (Table 1). Recruiter 1, who also had a Mastodon account with over 8300 followers, posted identical polls on both platforms, enabling platform comparisons. At the time, Recruiter 1 was the only recruiter with more than 8300 followers on Mastodon, which we deemed necessary for a reliable comparison of results.

**Table 1.** Overview of recruiters used for social media poll distribution.^a^

Recruiter	Followers (Twitter), n	Short description	Domain
Recruiter 1	~65,000	Professor	Physics
Recruiter 2	~39,000	Professor	Economics
Recruiter 3	~10,000	Medical researcher	Medicine
Recruiter 4	~2500	Professor	Psychology
Recruiter 5	~750	Professor	Computer science

a This table summarizes the accounts leveraged to recruit participants for the comparative study on COVID-19 infections. Included are the number of followers, short description, and domain. The recruitment targeted followers of these accounts in Germany between July 19 and July 26, 2023*.*

Polls were posted from July 19 to July 26, 2023 (7 days being the maximum due to platform limits) and included 2 multiple-choice questions on COVID-19 infection history. The polls were structured to allow respondents to select their answers or view results without participating.

A custom-built Twitter posting tool automated the posting process for 3 recruiters, ensuring consistent timing across accounts. Recruiters who posted manually followed similar guidelines to minimize timing bias. The polls were posted in a thread, with the last post being a post with a link to an external LimeSurvey questionnaire, allowing respondents to transition seamlessly from poll participation to survey completion. From now on, external survey always refers to the externally hosted LimeSurvey questionnaire that was linked at the end of the Twitter or Mastodon thread. All questions were originally formulated in German and translated for the purpose of this paper (see also Table S1 in Multimedia Appendix 1).

**Figure 1.**
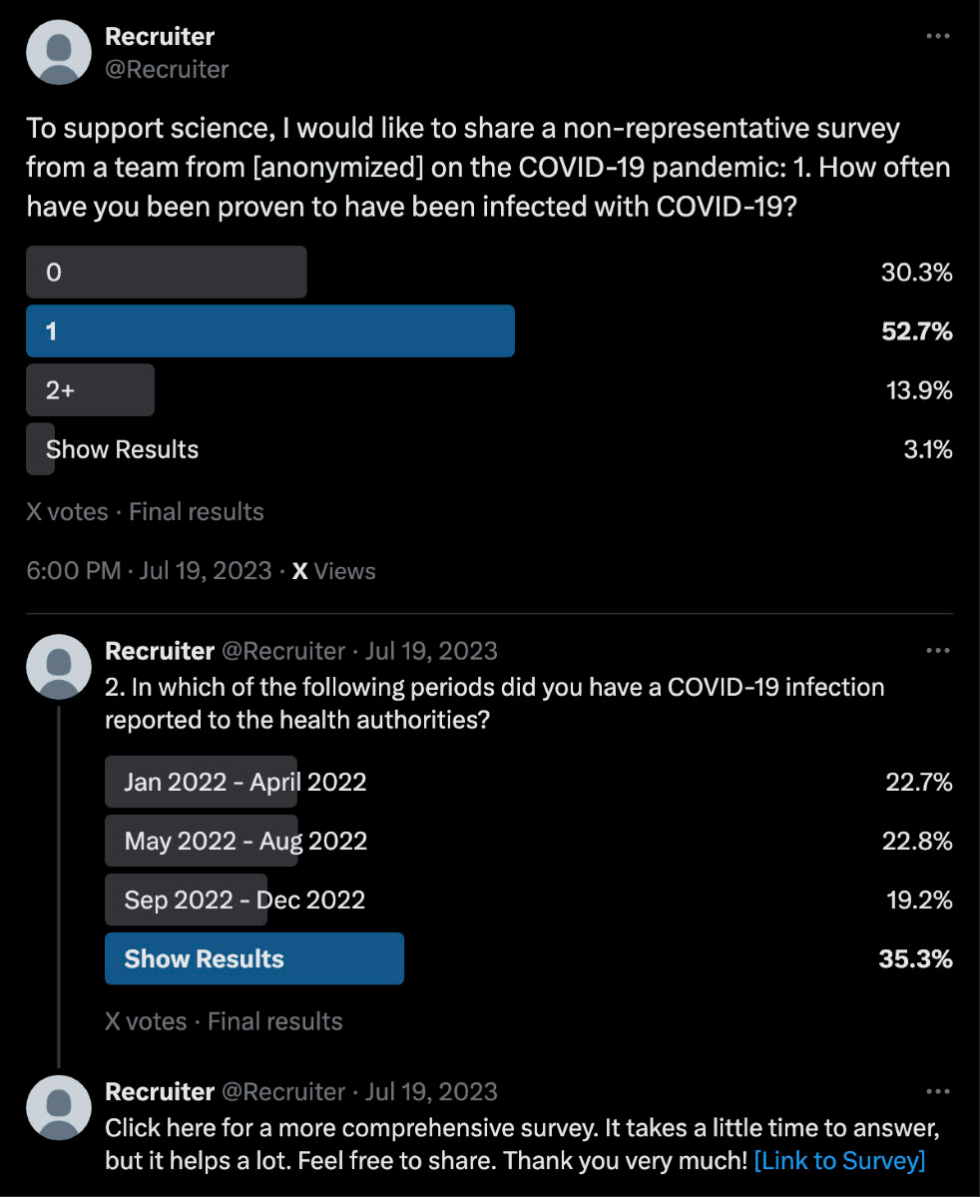
Example of a Twitter thread used for the comparative study on COVID-19. The figure illustrates the format and structure of a recruitment thread used to conduct poll-based data collection among any social media users who viewed the post, including followers of 5 recruiter accounts. All identifiable research information has been removed to maintain researcher anonymity. The recruitment took place in Germany between July 19 and July 26, 2023*.*

The initial social media post briefly explained the study purpose to ensure transparency and provide context. An anonymized example of the Twitter thread is shown in Figure 1, where identifiable details, including the location of the team and the survey link, have been removed.

### Data Collection

#### SMPs and External Survey

The external survey was open from July 18 to August 30, 2023. It collected detailed information on sociodemographics, health status (COVID-19 infections, vaccination history, preexisting conditions), and social contacts. The survey formed part of a broader research project and therefore contained several additional sections that are not examined in this paper. Because the overall instrument was comparatively long, we anticipated a substantial dropout rate. For this reason, the study design explicitly incorporated the use of incomplete questionnaires, provided that respondents had supplied sufficient data for the analyses reported here. Respondents were informed about data collection and processing at the start of the external study, and consent was explicitly requested. The participation requirements were only that participants have access to the survey link and be at least 18 years of age. All variables and the survey are available in the preregistration on OSF [48]. The contact data will be described here but analyzed in depth in a separate paper [49].

A summary of survey responses by medium is described in Table 2.

From the preregistration, we expected 5000 participants in the SMPs, which we exceeded by 1172 participants. For the external survey, we expected 500 responses. The 867 responses received and used for analysis exceeded our target. We also used incomplete responses for our analysis.

**Table 2.** Survey response data across social media platforms in the comparative study on COVID-19.^a^

Platform and question/type		Responses, n
Twitter		
Question 1		4370
Question 2		2129
Mastodon		
Question 1		1802
Question 2		738
External survey (excluding speeders^b^)		
Started		867
Completed		398
Survey shares		68
Completed shared surveys		12

a The table reports response counts from any social media users who answered polls posted via 5 recruiter accounts on Twitter and Mastodon, as well as participants in the external survey. Data are disaggregated by platform and question. Recruitment and data collection occurred in Germany between July 19 and July 26, 2023, for Twitter and Mastodon and between July 18 and August 30, 2023, for the external survey.

b A speeder is defined as a participant completing the survey in less than one-third of the median time.

All responses were anonymized during data processing. Personally identifiable information, such as IP addresses and free-text responses, was removed, and speeders were excluded from the analysis as per the definition in the preregistration [48]. It is assumed that participants falling into this category did not provide meaningful data points and therefore were excluded. Data preprocessing and analysis were performed using R version 4.4.1 and the *tidyverse* packages (version 2.0.0) [51,52]. The code to reproduce the analysis is publicly available on GitHub [53].

The average completion time was around 17 minutes. We did not check device compatibility, as our survey did not have any specific device requirements. No attention checks were included in the survey design. All participant answers that were not excluded for qualifying as speeders were used for the analysis. Missing data were not imputed (see supplementary materials in Multimedia Appendix 1 for detailed reporting of missing data by variable).

#### Multilocal and Serial Prevalence Study of Antibodies Against Respiratory Infectious Diseases in Germany

In the spring of 2022, a subset of 9921 of the invited 33,426 MuSPAD participants took part in the corresponding survey round. These 9921 participants are the source of all demographic data presented in this comparison. The next round of data collection was conducted in the winter of 2022/2023. This is the source of all data on the number of infections, the time of infection, and the number of vaccinations. Here, we only considered the responses of the 9921 participants who had already participated in spring 2022. However, not all participants responded to the winter survey, which reduced the sample size to 5128 participants.

### Data Processing

The MuSPAD data were merged from the independent waves using the following procedure: use the “user_id” column to assign results to participants and merge all results. MuSPAD collected the number of infections up to the end of 2022; participants could only report 1 date of infection for the period from April 1, 2023, to August 31, 2023. Therefore, incidence during this period was calculated based on a maximum of 1 reported infection per participant.

We apply weighted bootstrapping to ensure that the age distribution of participants in the external survey matches the age distribution of the German population (see *Demographic Comparison* section). This also allows for comparisons of the 7-day incidence per 100,000 between the external survey, the MuSPAD study, and the official reporting statistics by RKI. To compute the mean 7-day incidence and the empirical 95% CI of this mean for each point in time, we apply bootstrapping. We perform 1000 bootstrap iterations, resampling with replacement within each age group, with sample sizes weighted according to population age distributions, to generate incidence estimates.

### Comparison of Data Sources

We evaluated the representativeness and reliability of the data collected via SMPs and the external survey against the following conventional sources:

1. MuSPAD [28,29]: a sequential seroprevalence study of SARS-CoV-2 infections and vaccinations involving 5128 participants in 2022‐2023.
2. COSMO [30,54]: a cross-sectional serial online survey capturing public perceptions of COVID-19, with data from 1003 respondents in late 2022.
3. Official reports: 7-day incidence rates and vaccination statistics from RKI [55,56] and demographic data from the FSO collected on a daily basis.

For demographic comparisons, gender [57], age [58], household size [59], education level [60], and occupation [61] data were used, sourced from MuSPAD and national statistics. Age brackets were introduced into the data to facilitate comparison with other data. These consist of the age-in-years brackets: 18‐39, 40‐59, 60‐79, and 80‐99.

### Analysis Framework

Descriptive statistics were used to analyze the distribution of infection rates, vaccination history, and demographic data. Comparisons included differences between Twitter and Mastodon responses, as well as variations among the 5 recruiters. Furthermore, we excluded the “show results” votes on the SMPs from the analysis (“show results” means that on Twitter and Mastodon, users submitted no valid answer themselves but only looked at the votes of others, providing no data points).

To estimate differences in infection distributions, we used contingency tables and reported Cohen *w* as a measure of total sample differences. To compare infection timelines across data sources, we calculated pairwise Pearson and Spearman correlation coefficients and conducted corresponding correlation tests to obtain *P* values for each comparison. Data from the external survey were compared against MuSPAD and the FSO to quantify bias.

We computed 95% CIs for any sample proportion, $\hat{p}$. As we assumed a binomial distribution for the true population proportion and as the binomial distribution is approximately normal for large enough samples, we used *z* scores when computing the CIs.

## Results

### Summary of Results

We found similar results for all samples regarding infection frequency, timing of infections, number of vaccinations, 7-day incidences, and demographic differences, although some differences could be explained by different measurement time periods or differences in measurement methods. Based on a sample of 6127 responses on Twitter and Mastodon and 867 responses from the external survey, the self-reported frequency of infection aligned closely with conventional sources, such as MuSPAD. Across all 4 studies, 28% to 38% of respondents reported never being infected (Twitter 28%, Mastodon 38%, external survey 34%, MuSPAD 33%), 50% to 56% reported one infection (Twitter 55%, Mastodon 50%, external 56%, MuSPAD 56%), and 8% to 17% reported 2 or more infections (Twitter 17%, Mastodon 12%, external 10%, MuSPAD 12%). The differences compared to MuSPAD were significant but showed only small effect sizes (Cohen *w* for Twitter: 0.152, Mastodon: 0.188, and external survey: 0.105). We also compared the subsample of SMPs by recruiters, indicating that follower size did not strongly impact findings.

### COVID-19–Related Comparisons

Table 3 provides an overview of the demographic composition and self-reported COVID-19 infection history across the different samples. While the age distributions of Twitter and Mastodon participants are comparable, both differ from the MuSPAD dataset, which includes a broader age range. The proportion of self-reported infections is also relatively consistent across social media–based samples and MuSPAD, although participants from MuSPAD reported fewer repeat infections. These similarities and differences highlight the role of recruitment methods in shaping sample characteristics.

**Table 3.** Demographic characteristics and COVID-19 infection data comparing external survey participants with the Multilocal and Serial Prevalence Study of Antibodies Against Respiratory Infectious Diseases cohort.^a^

Characteristic	Recruited via Twitter (N=565)	Recruited via Mastodon (N=276)	MuSPAD^b^ (N=9921)	Overall (N=10,762)
Age (y)				
Mean (SD)	48.9 (11)	48.6 (10.1)	57.1 (16.5)	56.4 (16.2)
Median (minimum-maximum)	49 (18-83)	49 (21-75)	59 (19-101)	58 (18-101)
Missing, n (%)	8 (1.4)	5 (1.8)	111 (1.1)	124 (1.2)
Gender, n (%)				
Female	318 (56.3)	130 (47.1)	5966 (60.1)	6414 (59.6)
Male	242 (42.8)	135 (48.9)	3861 (38.9)	4238 (39.4)
No answer	4 (0.7)	6 (2.2)	79 (0.8)	89 (0.8)
Other	1 (0.2)	5 (1.8)	15 (0.2)	21 (0.2)
Number of COVID-19 infections, n (%)				
0	194 (34.3)	95 (34.4)	5730 (57.8)	6019 (55.9)
1	311 (55)	157 (56.9)	3474 (35)	3942 (36.6)
≥2	60 (10.6)	23 (8.3)	429 (4.3)	512 (4.8)
No answer	0 (0)	1 (0.4)	288 (2.9)	289 (2.7)

a This table contrasts participants recruited via Twitter and Mastodon with the MuSPAD dataset in terms of age, gender, and self-reported history of COVID-19 infection. The social media survey sample comprised followers of 5 recruiter accounts in Germany, with data collected between July 19 and July 26, 2023. The MuSPAD dataset serves as a conventional cohort reference to contextualize the representativeness of the social media–based sample.

b MuSPAD: Multilocal and Serial Prevalence Study of Antibodies Against Respiratory Infectious Diseases.

**Figure 2.**
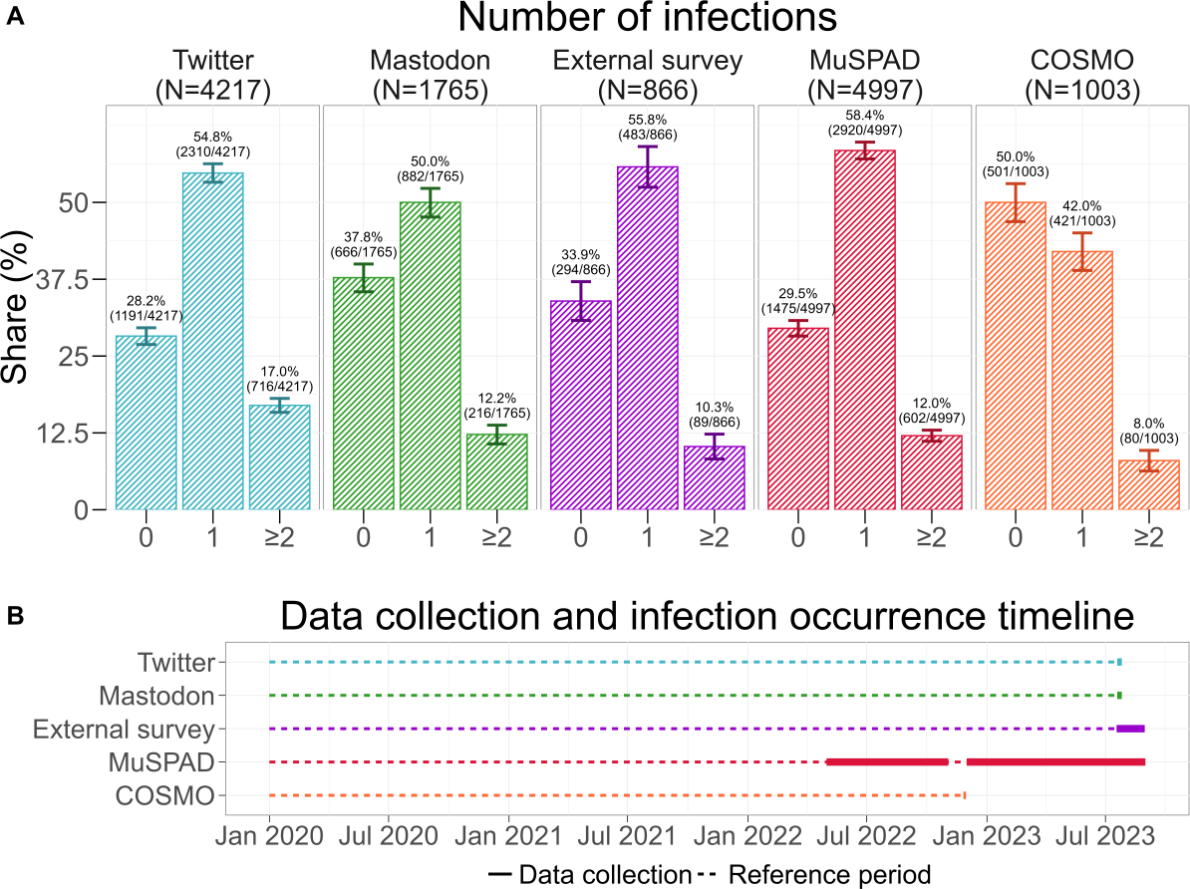
(A) Share of participants who reported having been infected 0, 1, or ≥2 times with COVID-19 since the beginning of the COVID-19 pandemic. Sample sizes reflect that not all participants provided an answer. Error bars represent 95% CIs (see *Analysis Framework* section for details). The visible differences between the COVID-19 Snapshot Monitoring (COSMO) study and the other 4 studies can be traced back to different data collection periods (see panel B). (B) Timeline depicting the different data collection periods. Bold blocks represent the time frame of actual data collection, and the dotted lines represent the time frame during which infections could have occurred. The COSMO study ended in November 2022 and thus does not include infections from 2023 onward. MuSPAD: Multilocal and Serial Prevalence Study of Antibodies Against Respiratory Infectious Diseases.

The results from Twitter, Mastodon, the external survey, and MuSPAD are largely consistent in terms of reported infection history (Figure 2). For these 4 studies, the proportion of respondents who stated that they had never been infected is comparable, varying between 28% and 38% (Twitter: 1191/4217, 28%, 95% CI 26.9%-29.6%; Mastodon: 666/1765, 38%, 95% CI 35.5%-40%; external survey: 294/866, 34%, 95% CI 30.8%-37.1%; MuSPAD: 1475/4997, 30%, 95% CI 28.3%-30.8%). Similarly, around half of the respondents of each study stated that they had been infected once (Twitter: 2310/4217, 55%, 95% CI 53.3%-56.3%; Mastodon: 882/1765, 50%, 95% CI 47.6%-52.3%; external survey: 483/866, 56%, 95% CI 52.5%-59.1%; MuSPAD: 2920/4997, 58%, 95% CI 57.1%-59.8%). The percentage of respondents who reported experiencing at least 2 infections ranged from 8% to 17%, while the highest share was recorded on Twitter at 17% (716/4217, 95% CI 15.8%-18.1%), followed by Mastodon at 12% (216/1765, 95% CI 10.7%-13.8%), the MuSPAD study at 12% (602/4997, 95% CI 11.1%-12.9%), and the external survey at 10% (89/866, 95% CI 8.3%, 12.3%).

Overall, the number of infections reported on Twitter showed a difference compared to the reference MuSPAD data with Cohen *w* 0.152, Mastodon with Cohen *w* 0.188, and the external survey with Cohen *w* 0.105. We additionally computed a sensitivity analysis, which indicated that, given the smallest sample size (n=866) and assuming *α*=.01 and power=0.95, the study could detect effects of Cohen *w* of at least 0.154. We opted for a post hoc sensitivity analysis instead of a power analysis, as we are mostly interested in determining the threshold for differences that we could have detected rather than determining the power retrospectively.

In contrast, a comparison with the COSMO study shows visible differences. In the most recent round of the COSMO study, conducted on November 29, 2022, and November 30, 2022, 50% of participants (501/1003) reported that they had never been infected, 42% of participants (421/1003) reported that they had been infected once, and 8% (80/1003) reported that they had been infected at least 2 times. Due to its implementation period, the COSMO study does not account for infections that occurred in 2023, impeding a meaningful comparison between the COSMO study and the other 4 studies. However, higher counts for zero infections seem reasonable at an earlier point in time. Summarizing, we can state that the number of infections was captured similarly between samples.

**Figure 3.**
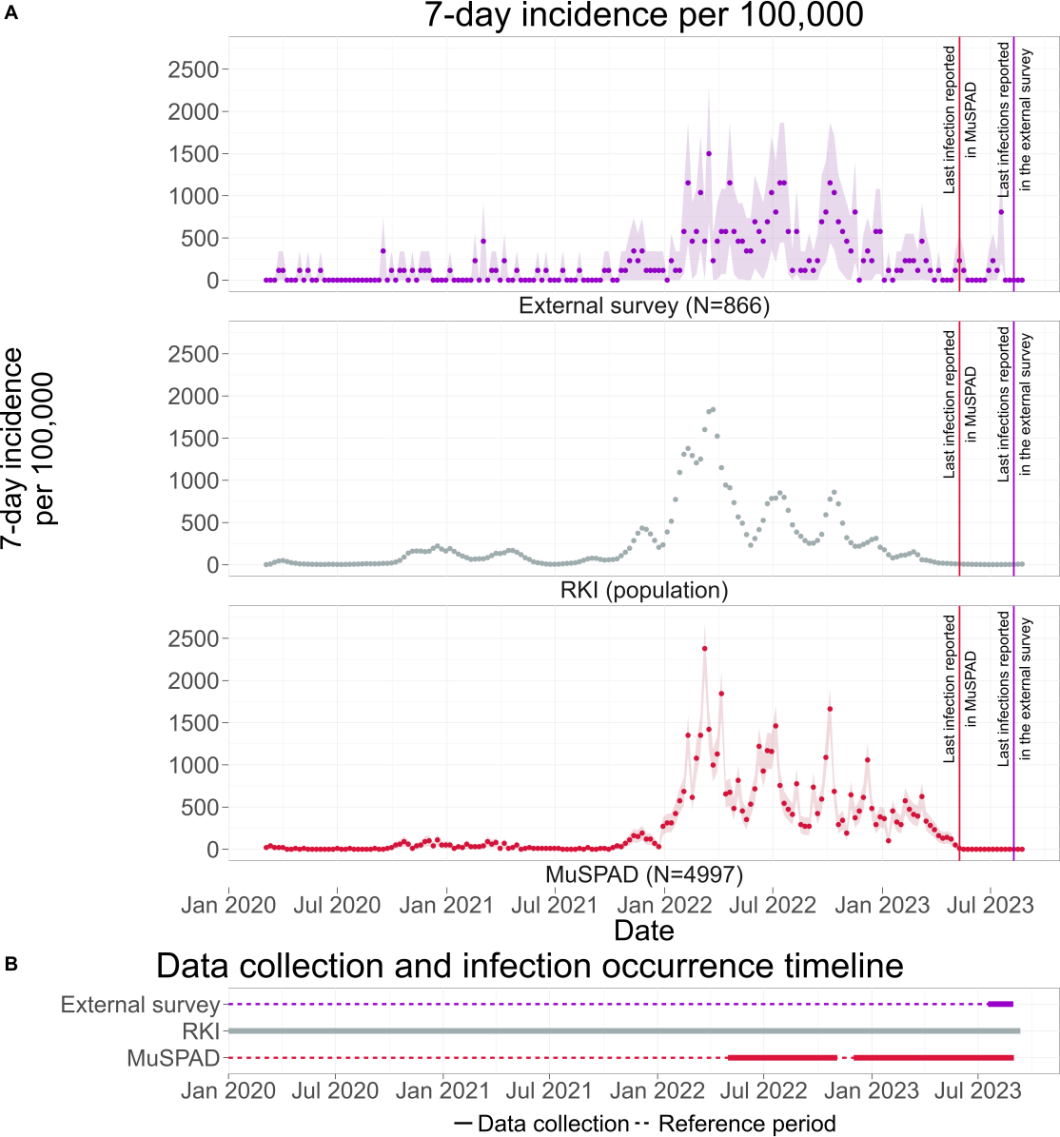
(A) 7-day incidence per 100,000 from March 2020 until summer 2023. For the external survey and the Multilocal and Serial Prevalence Study of Antibodies Against Respiratory Infectious Diseases (MuSPAD) study, the incidence for individuals aged 18 years and older is depicted, while for the Robert Koch Institute (RKI), the incidence for individuals aged 15 years and older is depicted. As the RKI does not provide case data in appropriate age bins, an exact match of age groups was not possible. The waves, local maxima, and local minima occur simultaneously in all 3 data sources. Ribbons represent 95% CIs (see *Analysis Framework* section for details). Bootstrapped results are presented in Figure S1 in Multimedia Appendix 1. For original German and English translations of corresponding survey items, see Table S3. (B) Timeline depicting the different data collection periods. Bold blocks represent the time frame of actual data acquisition, and the dotted lines represent the reference period, the time frame during which infections could have also occurred. In contrast to the external survey and the MuSPAD study, the RKI continuously collected infection data during the COVID-19 pandemic.

A comparison of the timing of infections (Figure 3) shows that the 7-day incidence per 100,000 from the external survey, the MuSPAD study, and the officially reported incidence by the RKI follow the same trend from March 2020 until data collection ended in summer 2023 (Twitter/Mastodon questions are excluded from the timing comparison as they do not allow the computation of a 7-day incidence per 100,000 for COVID-19 cases. See the *Data Collection* section for question formulations and Timing of Infection [Twitter and Mastodon] in the Supplementary for discussion of the votes on question 2.). Specifically, waves, local maxima, and local minima occur simultaneously in all 3 data sources. We further note that the local maxima in July 2022 and October 2022 are larger for the external survey and the MuSPAD study, reaching values around 1500, while the 7-day incidence per 100,000, according to the RKI, only reaches values around 500. Overall, patterns of infections are reasonably similar between samples, although measurements were conducted differently.

Spearman rank correlation analyses demonstrated strong positive associations between infection dates in the external survey and MuSPAD (ρ=0.884, *P*<.001) and between MuSPAD and RKI counts (ρ=0.831, *P*<.001). The correlation between the external survey and RKI counts was large as well (ρ=0.640, *P*<.001). Corresponding Pearson coefficients (0.839, 0.856, and 0.599, respectively) showed similar patterns of association. All correlations were statistically significant, indicating temporal concordance across the 3 data sources.

Both the external survey and the MuSPAD study undersample 1-person households. Since the external survey overrepresents 40- to 59-year-old people (see *Demographic Comparison* section), we applied bootstrapping to adjust the age distribution for comparability with RKI-reported 7-day incidence per 100,000 (see *Data Processing* section). As the adjustment had a negligible impact on trends, we present the raw data here, with bootstrapped results available in Bootstrapping in Multimedia Appendix 1.

**Figure 4.**
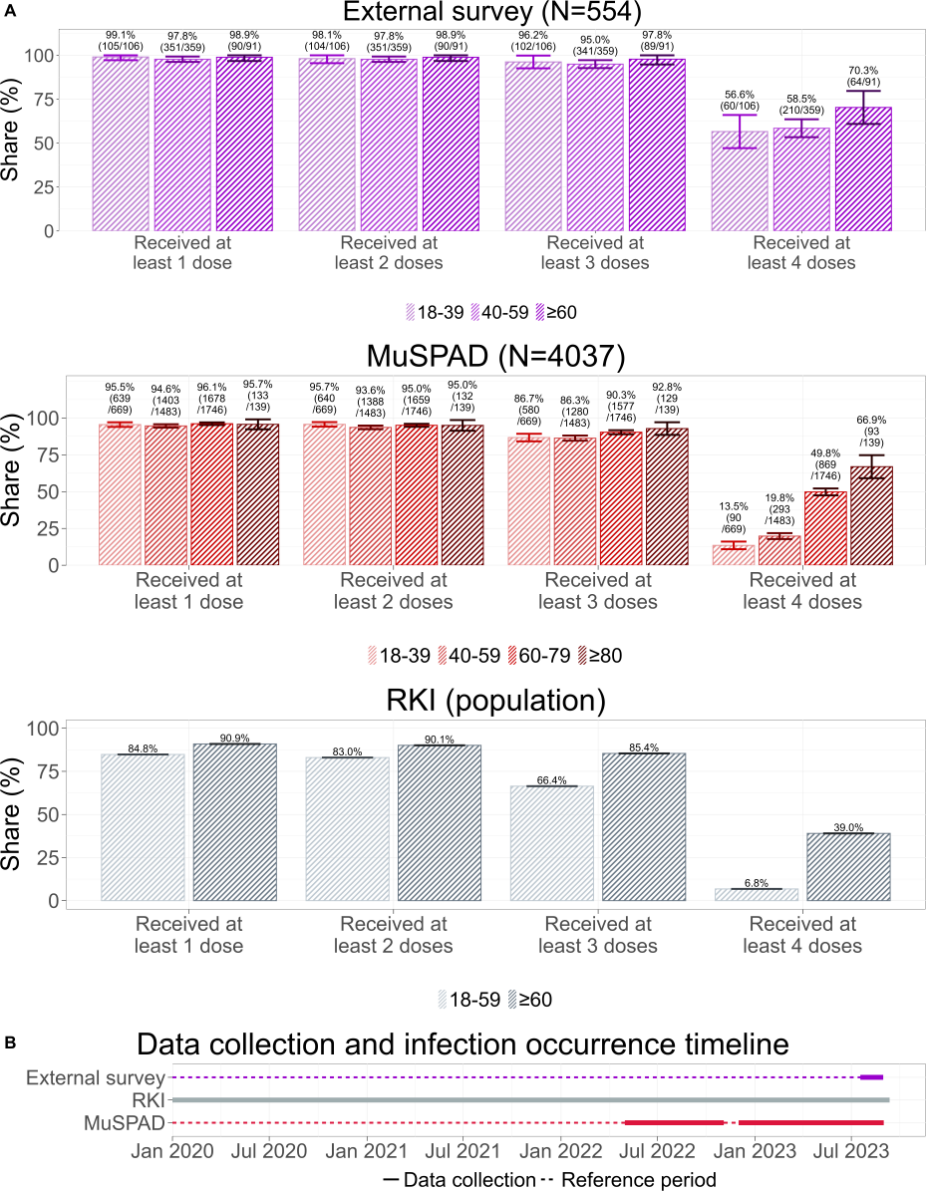
(A) Shares of individuals who have received at least 1, 2, 3, or 4 doses of any COVID-19 vaccination by September 11, 2023. Both the external survey and the Multilocal and Serial Prevalence Study of Antibodies Against Respiratory Infectious Diseases (MuSPAD) study failed to recruit large numbers of unvaccinated individuals and individuals who decided not to get or could not get a booster shot. Error bars denote 95% CIs (see *Analysis Framework* section for details); for Robert Koch Institute (RKI) data, these are negligible due to the large sample size. (B) This timeline illustrates the data collection periods for 3 different sources: the external survey, the RKI dataset, and the MuSPAD study. The solid bold segments indicate the actual data collection periods, while the dotted lines represent the corresponding reference periods—time frames during which vaccinations could have been administered and retrospectively reported. The alignment of these periods ensures consistency in comparing vaccination data across sources. The external survey and MuSPAD are retrospective data collections, while the RKI uses continuous data collection.

The analysis of the number of vaccination doses received until September 11, 2023, shows comparable results between the external survey and the MuSPAD study (Figure 4). That is, almost all participants received at least 2 doses of a COVID-19 vaccine of any brand, with no discernible differences between the 18- to 39-year-olds, 40- to 59-year-olds, 60- to 79-year-olds, and 80- to 99-year-olds. However, the MuSPAD study shows slightly lower percentages. In both the external survey and the MuSPAD study, we find a small drop between the share of participants who reported receiving at least 2 doses and those who reported receiving 3 doses for the 18 to 39, 40 to 59, and 60 to 79 years age groups, not distinguishing between homologous or heterologous booster shots. A notable difference between the 2 studies emerges in the share of participants who received at least 4 doses of the COVID-19 vaccine, although fourth dose recommendations were intended for older or at-risk populations. Across all age groups, apart from the 80 to 99 years age group, the external survey reports higher vaccination rates than the MuSPAD study.

The comparison of vaccination numbers between the 2 studies and the officially reported numbers by the RKI reveals 2 key differences. First, the RKI splits the adult population only into 2 age groups (18‐59 and 60+ years), thus limiting the comparability. Second, it can be noted that both the external survey and the MuSPAD study struggle to recruit unvaccinated individuals and individuals who decided not to or could not receive a third or fourth vaccine dose. However, the reported variable COVID-19 vaccination status showed a considerable amount of missing data (300/867, 34.6%), which might include unvaccinated individuals.

### COVID-19–Related Comparison by Recruiter

Analysis of responses to the first Twitter or Mastodon question reveals that most votes stem from Recruiter 1 and Recruiter 2 (5589/6172, ~90%), making them the dominant contributors to the Twitter sample (Table 4).

**Figure 5.**
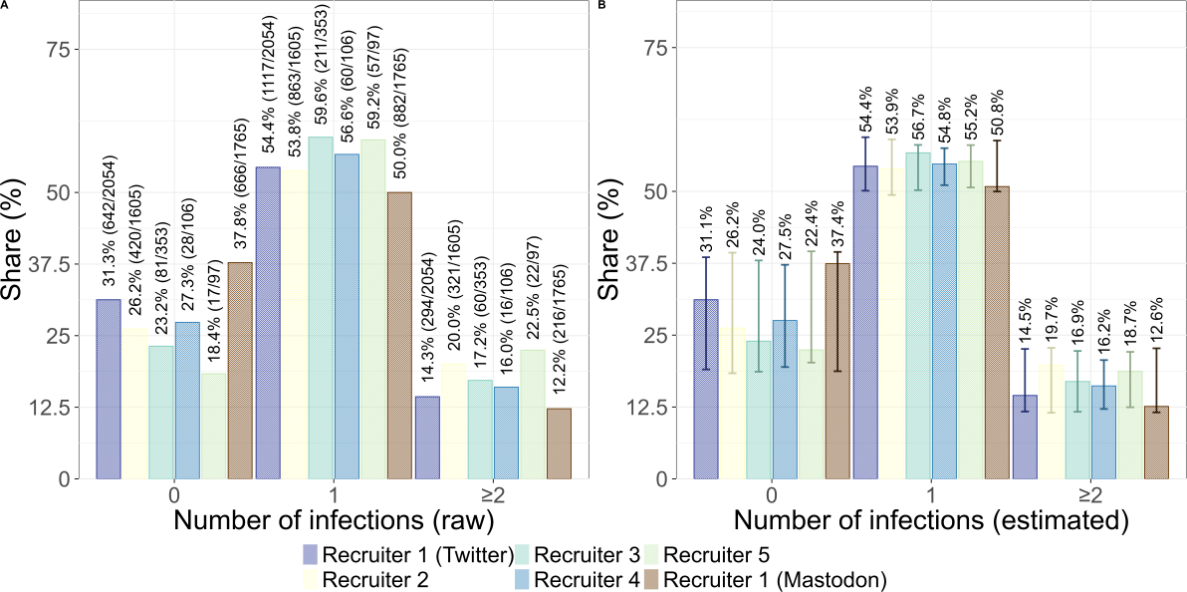
Raw and estimated proportions of participants reporting 0, 1, or ≥2 COVID-19 infections in the first social media poll, by recruiter account and platform. Estimates were created using a generalized linear model with the recruiter as a random intercept. The figure compares responses from participants recruited via Twitter and Mastodon. Overall patterns were similar across recruiters; however, the largest proportion of participants reporting zero infections came from the Mastodon-recruited group. Error bars were created using parametric bootstrapping (500 simulations) on mixed-effects logistic regression models to generate 95% CIs. Participants who selected “show results” were excluded from this analysis. Data were collected in Germany between July 19 and July 26, 2023*.*

Breaking down infection history by recruiter reveals variations in reported infection rates. The shares of the participants who reported zero infections on Twitter, for example, fluctuate between 18% (17/97, Recruiter 5) and 31% (642/2054, Recruiter 1 [Twitter]) (see Figure 5). On Mastodon, however, the share of participants who reported zero infections is higher (666/1765, 38%). A similar, though less pronounced, variation is observed among participants who reported one infection: 50% (882/1765) of participants recruited by Recruiter 1 (Mastodon), 54% (863/1605) of participants recruited by Recruiter 2, 54% (1117/2054) of participants recruited by Recruiter 1 (Twitter), 57% (60/106) of participants recruited by Recruiter 4, 59% (57/97) of participants recruited by Recruiter 5, and 60% (211/353) of participants recruited by Recruiter 3 reported one infection. Recruiter 5 recruited, with 23% (22/98), the largest share of participants who reported at least 2 infections. However, the limited number of responses from Recruiter 5’s poll had little impact on the overall Twitter sample distribution. In summary, all recruiters drew similar samples regarding total infections.

**Table 4.** Number of votes on the first social media poll, by recruiter account and platform.^a^

Recruiter	Number of votes (question 1 only)
Recruiter 1 (Twitter)	2120
Recruiter 2	1667
Recruiter 3	371
Recruiter 4	111
Recruiter 5	101
Recruiter 1 (Mastodon)	1802

a This table shows the distribution of responses across the 5 recruiter accounts. Only Recruiter 1 shared the poll on both Twitter and Mastodon, while the remaining four recruiters distributed the poll exclusively on Twitter. Data were collected in Germany between July 19 and July 26, 2023.

### Demographic Comparison

**Figure 6.**
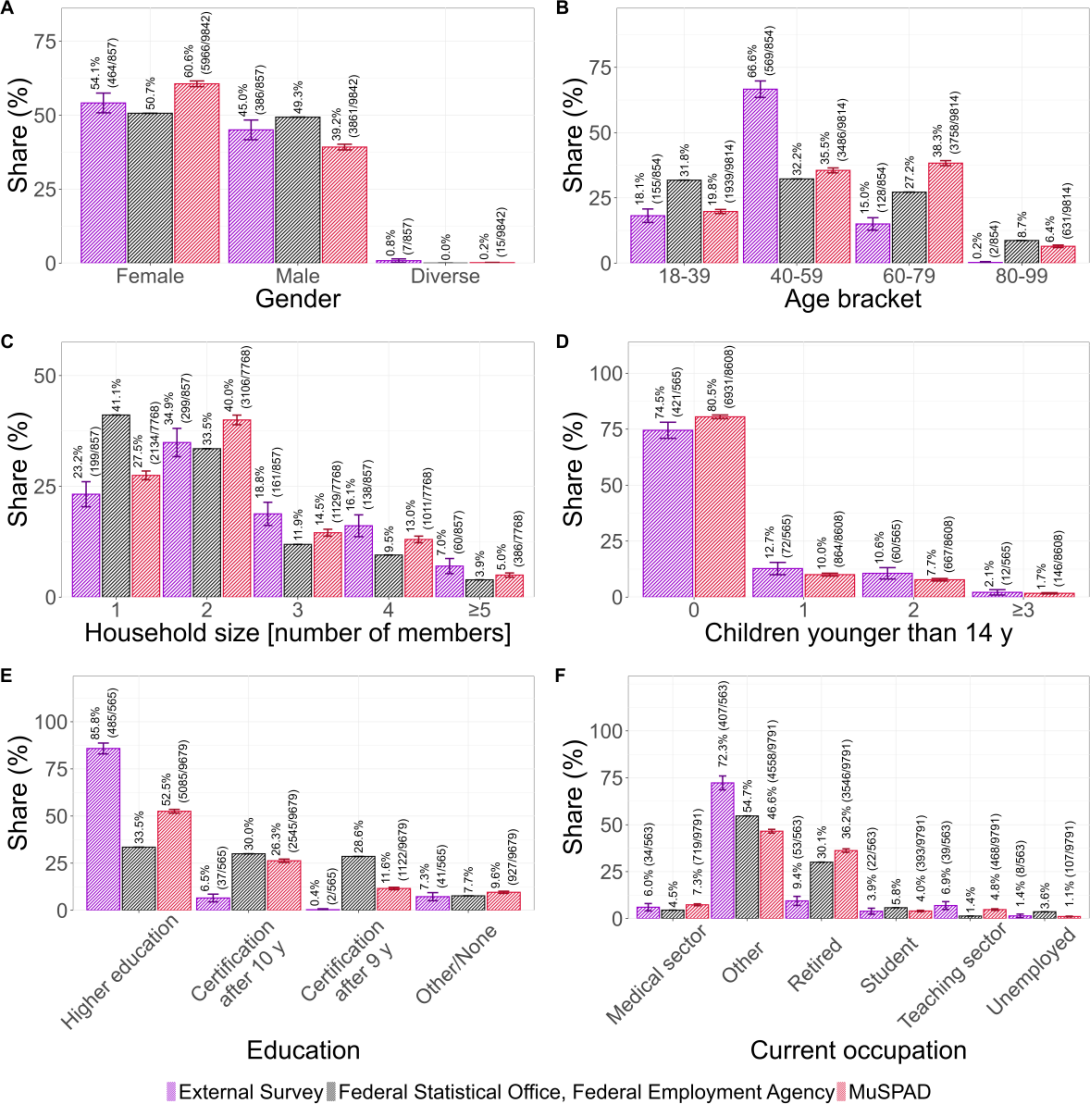
Comparison of sociodemographic distributions between the external survey, the Multilocal and Serial Prevalence Study of Antibodies Against Respiratory Infectious Diseases (MuSPAD) study, and the Federal Statistical Office (FSO) and Federal Employment Agency. Panel A shows the gender, panel B the age brackets, panel C the household size, panel D the number of children under 14 in the household, panel E the education level, and panel F the current occupation of participants. Participants who failed to answer the corresponding survey item were excluded from the analysis. Error bars represent 95% CIs (see *Analysis Framework* section for details); for FSO data, these are negligible due to the large sample size. Underage participants were excluded from the analysis, as neither the external survey nor the MuSPAD study recruited them (see panel B). Additionally, the FSO does not provide data on children younger than 14 years (panel D).

A demographic comparison of the external survey and the MuSPAD study with official statistics reveals notable deviations on various levels. That is, females are slightly overrepresented in the external survey but less so than in the MuSPAD study (external survey: 464/857, 54%, MuSPAD: 5966/9842, 61%, FSO: 51%; Figure 6A). Consequently, males are slightly underrepresented in both studies (external survey: 386/857, 45%, MuSPAD: 3861/9842, 39%, FSO: 49%), while respondents who indicated their gender as diverse make up less than 1% of both study’s samples (the FSO only distinguishes between “female” and “male”). In addition, participants aged 40 to 59 years are substantially overrepresented in the external survey (external survey: 569/854, 67%, MuSPAD 3486/9814, 36%, FSO: 27%). In contrast, individuals aged 80 to 99 years are underrepresented in both the external survey and the MuSPAD study (external survey: 2/854, <1%, MuSPAD: 631/9814, 6%, FSO: 9%; Figure 6B). Both the survey and the MuSPAD study undersample 1-person households (external survey: 199/857, 23%, MuSPAD: 2134/7768, 28%; Figure 6C), which make up 41% of households in Germany according to the FSO. Consequently, larger household sizes are slightly overrepresented. Furthermore, most external survey and MuSPAD participants (external survey: 421/565, 75%, MuSPAD: 6931/8608, 81%) replied that they had no children under the age of 14 years (Figure 6D). One (external survey: 72/565, 13%, MuSPAD: 864/8608, 10%) and 2 (external survey: 60/565, 11%, MuSPAD: 667/8608, 8%) children under the age of 14 years are similarly likely in both studies, while only 2% (external survey: 12/565, 2%, MuSPAD: 146/8608, 2%) of respondents reported having 3 or more children under the age of 14 years. Participants who have received higher education are overrepresented in the external survey (external survey: 485/565, 86%, MuSPAD: 5085/9679, 52%, FSO: 34%). Thus, only 7% (37/565) reported they had obtained a certification after 10 years (MuSPAD: 2454/9679, 26%, FSO: 30%), and less than 1% (2/565) reported that they had obtained a certificate after 9 years (MuSPAD: 927/9679, 12%, FSO: 29%; Figure 6E). Finally, the external survey oversampled participants who reported their current occupation as “other” (external survey: 407/563, 72%, MuSPAD: 4558/9791, 47%, Federal Employment Agency: 55%), while it undersampled retired participants (external survey: 53/563, 9%, MuSPAD: 3546/9791, 36%, Federal Employment Agency: 30%; ).

To conclude, both novel sampling methods (eg, external survey, SMPs) introduce biases regarding demographic data.

## Discussion

### Principal Findings

In the time period assessed here—3 years into a coronavirus pandemic and after the establishment of large testing schemes in the population—the data collected via SMPs mirror cumulative self-reported infections in the assessed epidemic panel. In particular, it matches the proportion of people reporting having had infections. This shows the potential of SMPs, which can be an adequate and cost-effective proxy for infection numbers in such situations and can provide rapid and readily available data for dynamic modeling. Epidemic panels are particularly valuable during pandemic situations, as they enable serological estimates of cumulative infection prevalence and real-time monitoring of infection rates, helping to identify potential underreporting of self-reported diagnoses. This applies to both early pandemic phases (before large-scale testing is available) and later intrapandemic periods (after interest in testing has declined), as well as to seasonal infections commonly monitored in epidemic panels, such as influenza, respiratory syncytial virus, or vector-borne diseases, where diagnosed cases often underestimate actual infections, particularly across different age groups.

If the focus is on the cumulative number of infections during mid-intrapandemic periods, near real-time and straightforward data collection methods, such as SMPs and the external survey, can provide rapid, directional insights, showing only small differences compared to larger serological studies like MuSPAD. Incidence gathered through recruitment via social media for an external survey is also strongly correlated with official data from public health authorities. While it is notable that our sample can produce such timely results at minimal cost, claims about reliability and representativeness should be tempered, acknowledging that they are limited to pandemic scenarios with similar disease characteristics such as COVID-19.

On the other hand, demographic comparisons reveal that 40‐ to 59-year-olds and individuals with higher education are overrepresented in the data, while 1-person households are underrepresented. The higher education level is expected because all recruiters are part of academia. Due to legal reasons, participants under 18 years could not participate in the SMP, which is a drawback compared to the official testing conducted by the RKI.

While these sample biases and statistically significant differences in infection numbers across different platforms did exist, effect sizes were small, indicating that SMPs could be useful in ascertaining infection trends in the population in a rapid and cost-effective way.

### Comparison With Prior Work

Similar to what Schober et al [33] suggest, SMPs might be used as additional, cost-effective interim or on-top data collection tools to enrich official data. We go beyond what Vidal-Alaball et al [47] achieved in their study, focusing on collecting data instead of evaluating public opinion. As Zhao et al [36] point out, we did consider bias in our data and explicitly tested for this, observing inherently higher education levels in the Twitter data compared to other survey types. While social media data have previously been used for opinion mining and behavioral studies in public health, SMPs have not been systematically applied to directly collect self-reported infection or incidence data.

In terms of comparison to serological indicators of cumulative infections at certain time periods, the presented data on self-reported infections are in line with published data from the IMMUNEBRIDGE project, of which the MuSPAD cohort was one part [62]. Comparison with COSMO data revealed that, due to the earlier data collection period of COSMO, infection numbers were lower compared to our data and MuSPAD, possibly indicating that a large portion of first infections occurred in 2023.

Data until 2022 showed that survey-based incidence aligned closely with the officially reported figures from the RKI. However, a discrepancy emerged with the onset of the Omicron BA.5 wave in the summer of 2022. This could be due to individuals confirming infections with rapid antigen tests that were not reported or because many experienced only mild symptoms and did not seek medical attention, thus not entering official statistics. SMPs here allow accounting for this decline in official testing. Additionally, a potential limitation was that in the MuSPAD data, reported infections in the time frame April 1, 2023, to August 31, 2023, were limited to a single date, excluding possible secondary infections. Due to this, infection numbers during that time frame might be underreported.

Recall bias might have affected the reported timing of infections. shows that the incidence still aligns quite well with official reporting, indicating that the effect of recall bias was small overall.

Regarding vaccination doses, our data align well with the MuSPAD data except for the fourth vaccination dose. Here, our limited sample size regarding older age groups (60+) might be problematic because the fourth vaccination is recommended by the RKI only for these older age groups. Overall, compared to the RKI data, our sample and the MuSPAD sample generally have a higher proportion of vaccinated individuals. This might result from sampling bias, as very risk-averse and protective people might have a higher tendency to participate in a survey regarding a potentially dangerous infectious disease. We also report a high number of missing data points for the COVID-19 vaccination status, which might include unvaccinated individuals who do not want to report their status due to social desirability effects. The effect being present in the MuSPAD data also shows the difficulty in mitigating these effects even in high-effort, high-cost studies.

For the successful recruitment of a large enough sample size, a sizable followership on social media is required. Also, demographic comparisons have shown that biases exist when recruiters are not diverse regarding age, education, and background. Public health–related SMPs are more feasible in the later stages of a pandemic, when tests are widely available to the public and sufficient sentinel studies have already been conducted.

Overall, SMPs can complement traditional methods by providing real-time insights but cannot replace them due to data quality and representativeness concerns.

### Limitations and Future Work

Finally, it should be noted that there are limitations to SMPs: one does not obtain a list of the users who participated in the survey, but solely the proportion of responses for each option. Thus, this method cannot record demographic attributes, and subanalysis for specific target groups is impossible. Furthermore, only a subset of the population is active on Twitter, limiting the generalizability of these findings.

Another limitation of our study is the very small number of unvaccinated individuals included in the sample (external survey: n=10, 1.76%; MuSPAD: n=489, 3.63%), although the large number of participants who did not provide an answer here might include unvaccinated individuals.

Using 5 academically skewed accounts for recruitment generated 36,416 total Twitter impressions but introduced selection bias through extreme concentration (2 accounts controlled 87% of reach) and network homophily (followers share similar educated, professionally engaged demographics). Response rates of 17% to 19% indicate high engagement; however, the 56% drop in impressions and the 53% drop in votes between Question 1 and Question 2 reveal limitations in data gathering. Mastodon provided 2540 votes, but no impression data were available due to platform limitations, which prevented the calculation of response rates. This recruitment strategy systematically excludes individuals outside academic networks, thereby limiting the findings to “engaged followers of academic social media accounts” rather than more generalizable populations. Additionally, not all recruiters generated enough responses to detect small effect sizes when comparing them to each other. Although the number of infections shows variation, small sample sizes limit any meaningful analysis here.

Due to the limited data collected via Mastodon, H2 could not be fully answered. The Mastodon data, provided solely by Recruiter 1, included a higher proportion of single-person households compared to the overall sample, which may help explain the greater number of participants reporting no infections.

Future work includes improving the representativeness of SMPs, possibly through targeted outreach via ads on Twitter or Facebook, the inclusion of more platforms for data collection (eg, Threads, Bluesky, Facebook), and generating longitudinal data through weekly sampling via a proposed Twitter poll posting tool. This posting tool automatically posts polls every week or 2 weeks to inquire about infections, offering a highly cost-efficient method and allowing for automatic weekly evaluations. Additionally, to test the reliability of this method, a study with in-person testing could be run in parallel.

### Conclusions

Especially for recruitment, SMPs show promising results. We were able to generate a sizable sample, although it must be mentioned that this depends heavily on platform follower numbers. We showed that SMPs are an adequate and cost-effective tool for rapidly collecting health data. While overall demographic representativeness is good, discrepancies in age and education may impact generalizability. Especially for health data—in this case, infection numbers and incidence—data quality is comparable to that of more costly and high-effort panel studies. To respond to pandemic scenarios with similar disease characteristics such as COVID-19, data collected via SMPs can quickly provide accurate enough data to help with modeling efforts.

## Supplementary material

10.2196/80311 Multimedia Appendix 1 Supplementary materials showing further data on bootstrapping, timing of infection, demographic comparisons by Twitter/Mastodon recruiter, and comparisons of vaccine suppliers.

## Abbreviations

COSMO: COVID-19 Snapshot Monitoring
FSO: Federal Statistical Office of Germany
MuSPAD: Multilocal and Serial Prevalence Study of Antibodies Against Respiratory Infectious Diseases
NPI: nonpharmaceutical intervention measure
RKI: Robert Koch Institute
SMP: social media poll
STROBE: Strengthening the Reporting of Observational Studies in Epidemiology
WHO: World Health Organization
