# Supplementary material for: Rapid Epidemiological Data Collection on Social Media for COVID-19: Comparative Study Between Online Surveys and Conventional Cohorts
Source: J Med Internet Res. 2026 Apr 27;28:e80311. doi: 10.2196/80311 (PMC13117218; doi:10.2196/80311)
Supplement: Multimedia Appendix 1 [file jmir-v28-e80311-s001.docx]

# Bootstrapping


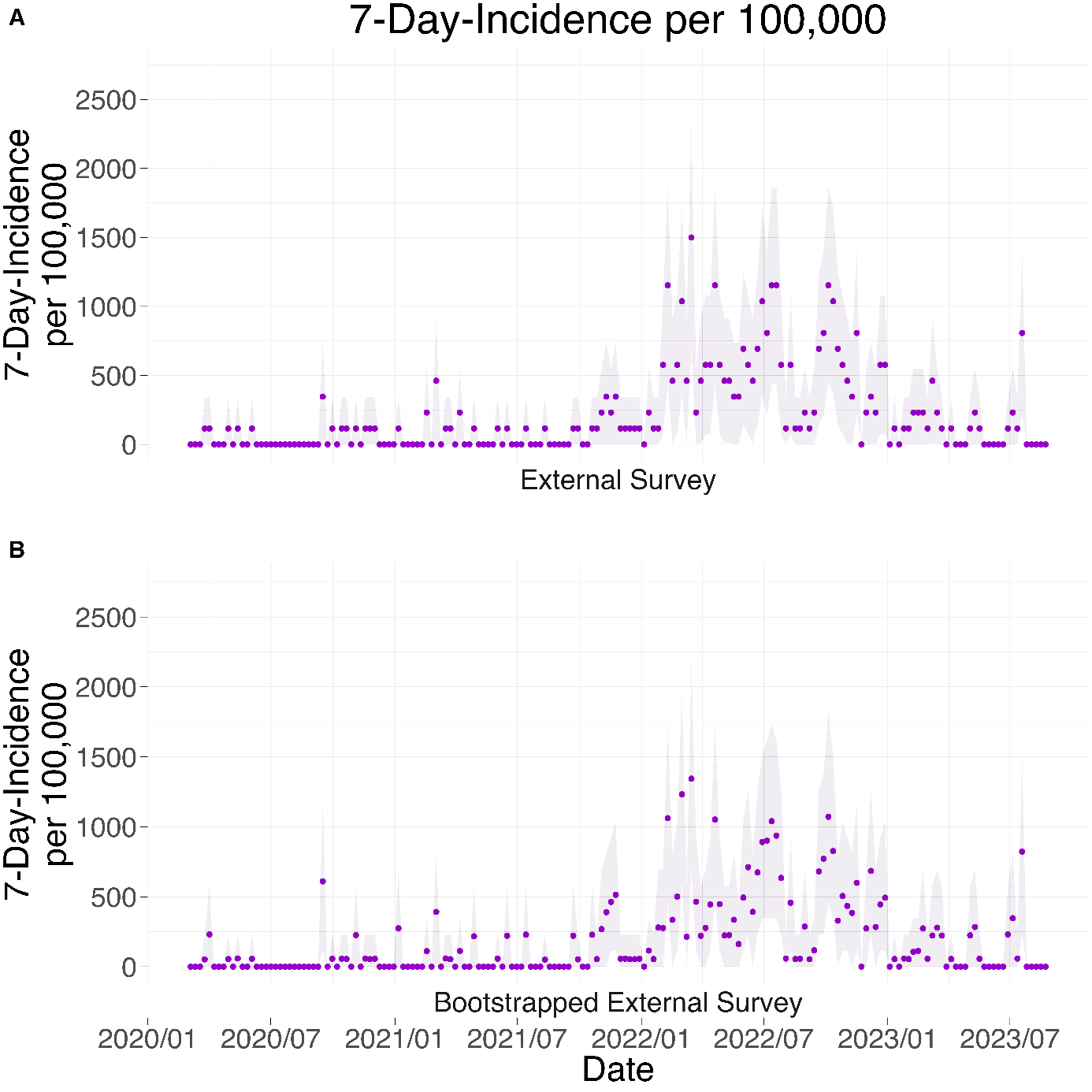


**Figure S1: A.** 7-day COVID-19 incidence per 100,000 estimated from the external survey sample collected as part of the comparative study of social-media-based surveillance. Ribbons show 95% confidence intervals (see Analysis Framework). **B.** Corresponding 7-day COVID-19 incidence after bootstrapping, with 95% of bootstrap samples falling within the ribbons. Participants were social media users in Germany who accessed the external survey between July 18 and August 30, 2023.

# Timing of Infection (Twitter and Mastodon)

| **Recruiter** | **Number of Votes (Question 2 only)** |
| --- | --- |
| Recruiter 1 (Twitter) | 1,131 |
| Recruiter 2 | 764 |
| Recruiter 3 | 172 |
| Recruiter 4 | 39 |
| Recruiter 5 | 23 |
| Recruiter 1 (Mastodon) | 738 |

***Table S1:*** *Number of votes on the second poll question on Twitter and Mastodon in the comparative COVID-19 study. Any social media users in Germany who viewed the poll were able to vote. Only Recruiter 1 shared the question on Mastodon; the other four shared it on Twitter. Data were collected between July 19 and July 26, 2023.*


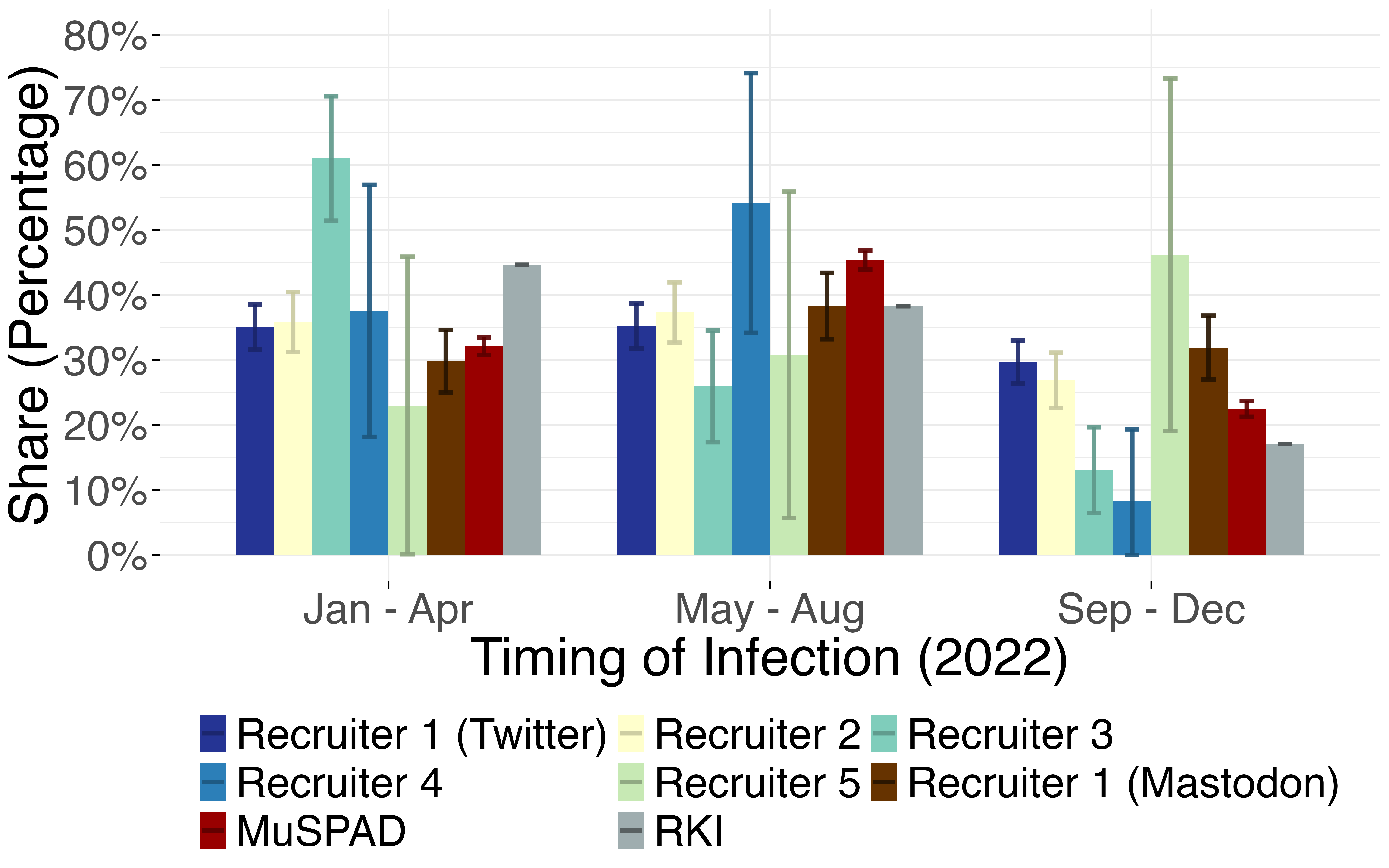


**Figure S2:** Shares of participants who indicated that they had reported a COVID-19 infection to public health authorities in Jan – Apr 2022, May – Aug 2022, Sep – Dec 2022 according to each Twitter recruiter and the one Mastodon recruiter. This question does not allow Twitter/Mastodon participants to report more than one infection in 2022, and participants who voted “Show results” were excluded from this analysis. For the RKI and MuSPAD bars, we counted all infections that were reported in 2022, binned them into the three time frames, and computed their shares. Here, it cannot be ensured that participants reported only one infection in 2022. Error bars represent 95% confidence intervals (see Analysis Framework for details).

On the second Twitter/Mastodon question, around a third of the participants recruited by Recruiter 1 (Twitter), Recruiter 2, Recruiter 4, and Recruiter 1 (Mastodon) voted that they had been infected between January and April 2022 (Fig. [8](#supfigure:TimingInfTwitter)). Exceptions are Recruiter 3, on whose poll more than 60% voted that they had been infected between January and April 2022, and Recruiter 5, on whose poll less than 25% voted that they had been infected in the first third of 2022. Note, however, the small number of votes on Recruiter 5’s poll. The results of the MuSPAD study are similar to the results of the Twitter/Mastodon question, while 45% of the infections that were reported to the RKI in 2022 were already reported in the first third of the year. For Recruiter 1 (Twitter), Recruiter 2, Recruiter 3, Recruiter 4, and Recruiter 1 (Mastodon), smaller shares voted that they got infected between September and December 2022. These results align with the shares according to the MuSPAD study and the RKI. The only exception is again Recruiter 5, on whose poll more than 45% of recruits voted that they got infected between September and December 2022.

# Demographic Comparison by Twitter/Mastodon Recruiter

| **Recruiter** | **Number of external survey participants** |
| --- | --- |
| Recruiter 1 (Twitter) | 387 |
| Recruiter 2 | 103 |
| Recruiter 3 | 60 |
| Recruiter 4 | 10 |
| Recruiter 5 | 4 |
| Recruiter 1 (Mastodon) | 264 |

***Table S2:*** *Number of participants who at least partially completed the external survey in the comparative COVID-19 study, categorized by their originating Twitter/Mastodon recruiter. The study targeted any social media users in Germany who accessed the external survey link between July 18 and August 30, 2023. Speeders (see Data Collection), unmatched participants, and those who accessed the survey through forwarded links rather than the recruiter accounts were excluded.*

Splitting the external survey participants by recruiter and comparing their sociodemographic attributes, we observe that Recruiter 1 (Twitter), Recruiter 3, and Recruiter 4 all over-recruited participants who reported their gender as female, while Recruiter 2 and Recruiter 5 under-recruited them (Fig. [9](#supfigure:DemographicComRecruiter1) A). Recruiter 5 mainly recruited participants between the ages of 18 and 39, and the other four mainly from the age group between 40 and 59 (Fig. [9](#supfigure:DemographicComRecruiter1) B). Regarding household size, Recruiter 5 recruited the largest share of 1-person- and 5+-person-households (Fig. [9](#supfigure:DemographicComRecruiter1) C). All recruiters over-recruited participants who reported receiving higher education (Figure [10](#supfigure:DemographicComRecruiter2) B).


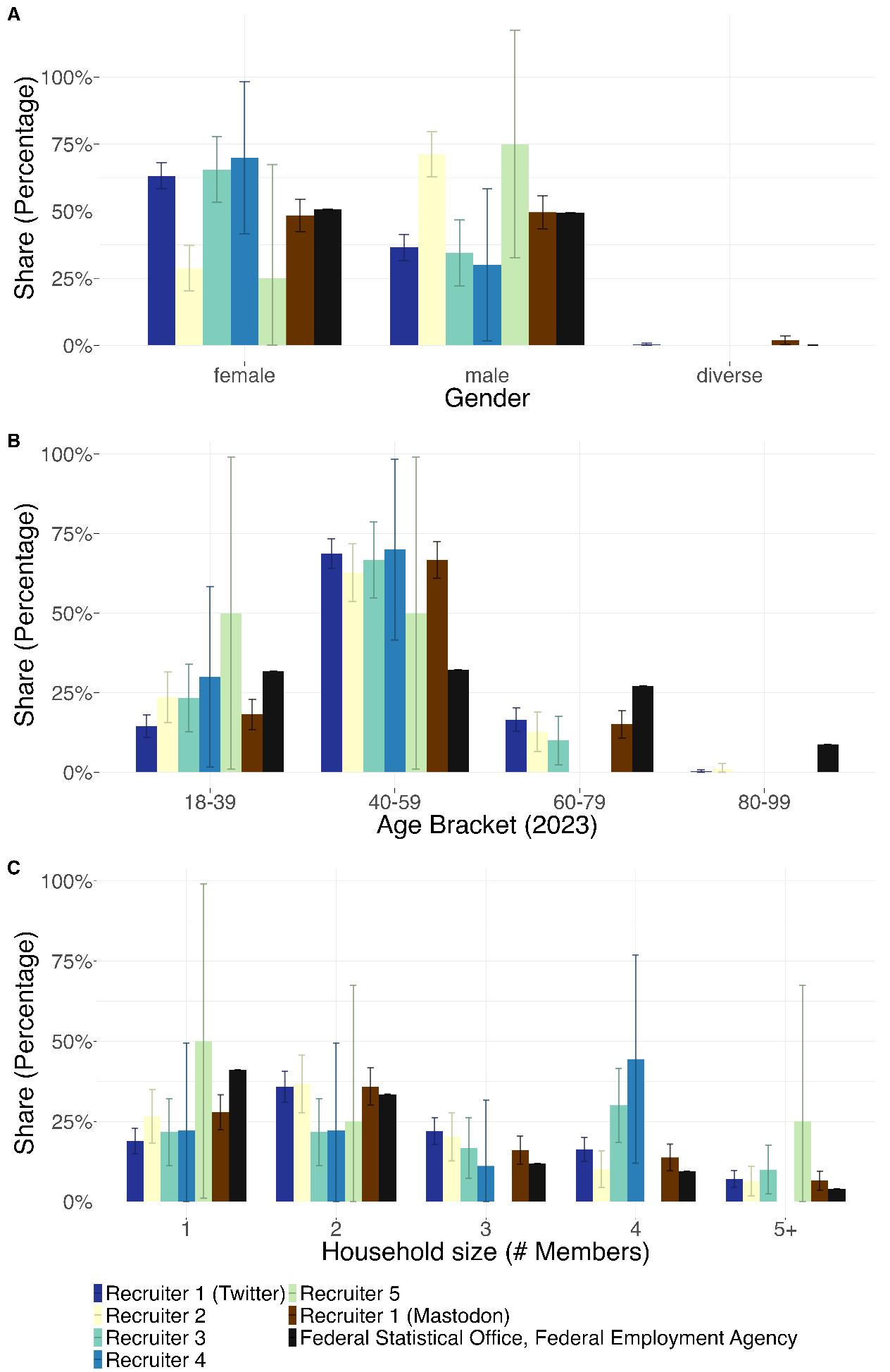


**Figure S3:** Comparison of gender, age, and household size among participants in the comparative COVID-19 study, contrasting the five Twitter recruiters and one Mastodon recruiter with official statistics from German national agencies (Federal Statistical Office/Employment Agency). Participants represent any social media users in Germany who accessed the external survey between July 18 and August 30, 2023. Error bars reflect 95% confidence intervals (see Analysis Framework).


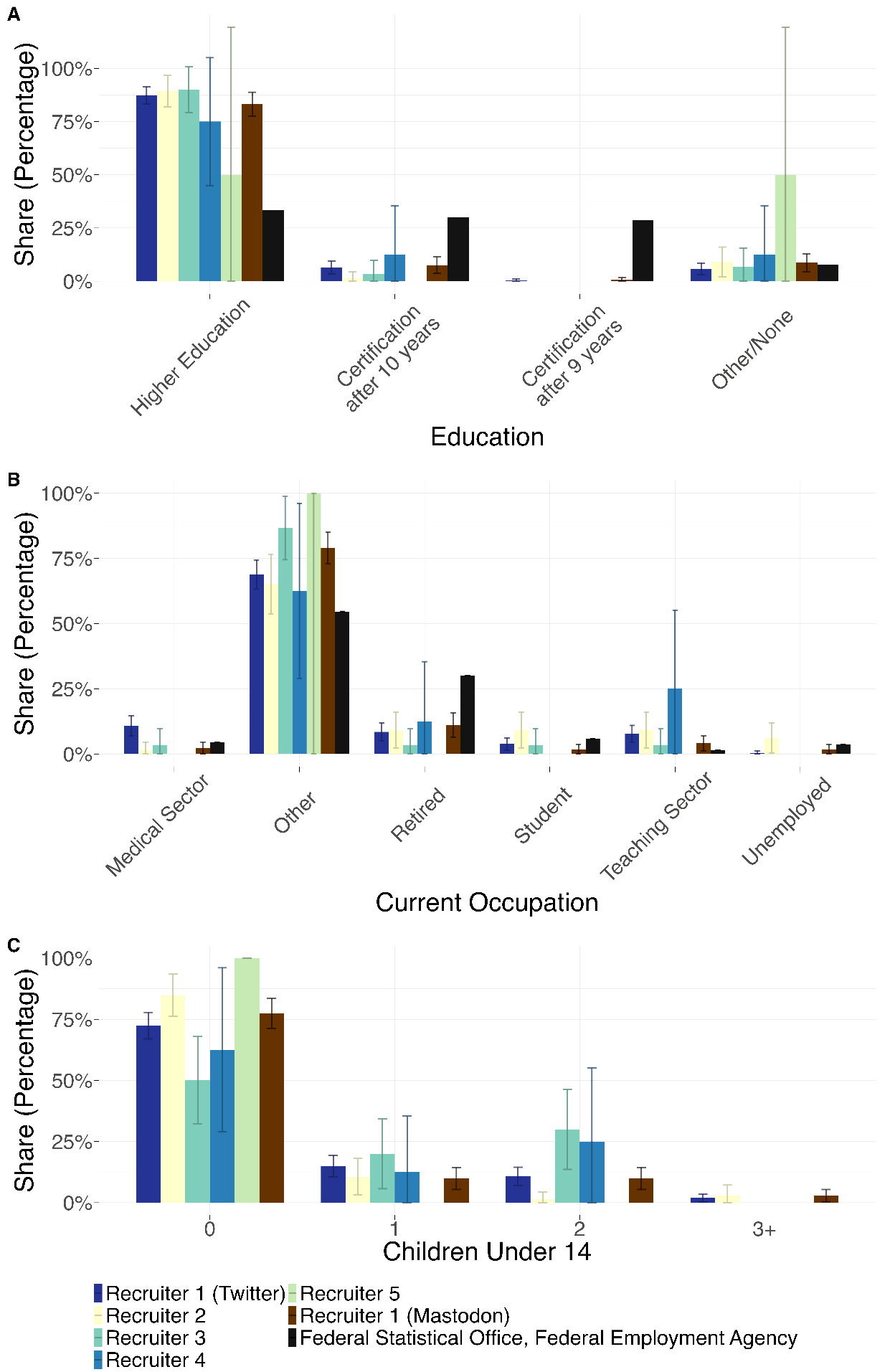


**Figure S4:** Comparison of the number of children under the age of 14, education, and current occupation between the five Twitter recruiters, the one Mastodon recruiter, and official reporting by the Federal Statistical Office/Employment Agency. Participants who failed to answer the corresponding external survey item were excluded from this analysis. Error bars represent 95% confidence intervals (see Analysis Framework for details). Participants were any social media users in Germany responding to the external survey between July 18 and August 30, 2023.

# Comparison of Vaccine Suppliers





**Figure S5**: Vaccination suppliers for different COVID-19 vaccine doses as reported by participants of the comparative study. Data originate from any social media users in Germany who accessed the external survey between July 18 and August 30, 2023. Error bars represent 95% confidence intervals.

For all four vaccination doses, RKI reported that a slightly larger share of vaccinated individuals had received the vaccine by BioNTech [56] than in the external survey and the MuSPAD study (Fig. [11](#supfigure:Vaccination_Supplier)). Both in the external survey and in the MuSPAD study, as well as for all four vaccination doses, a larger share of participants reported that they had received Moderna’s vaccine than according to the RKI. Apart from the aforementioned deviations, the results across studies and doses are comparable.


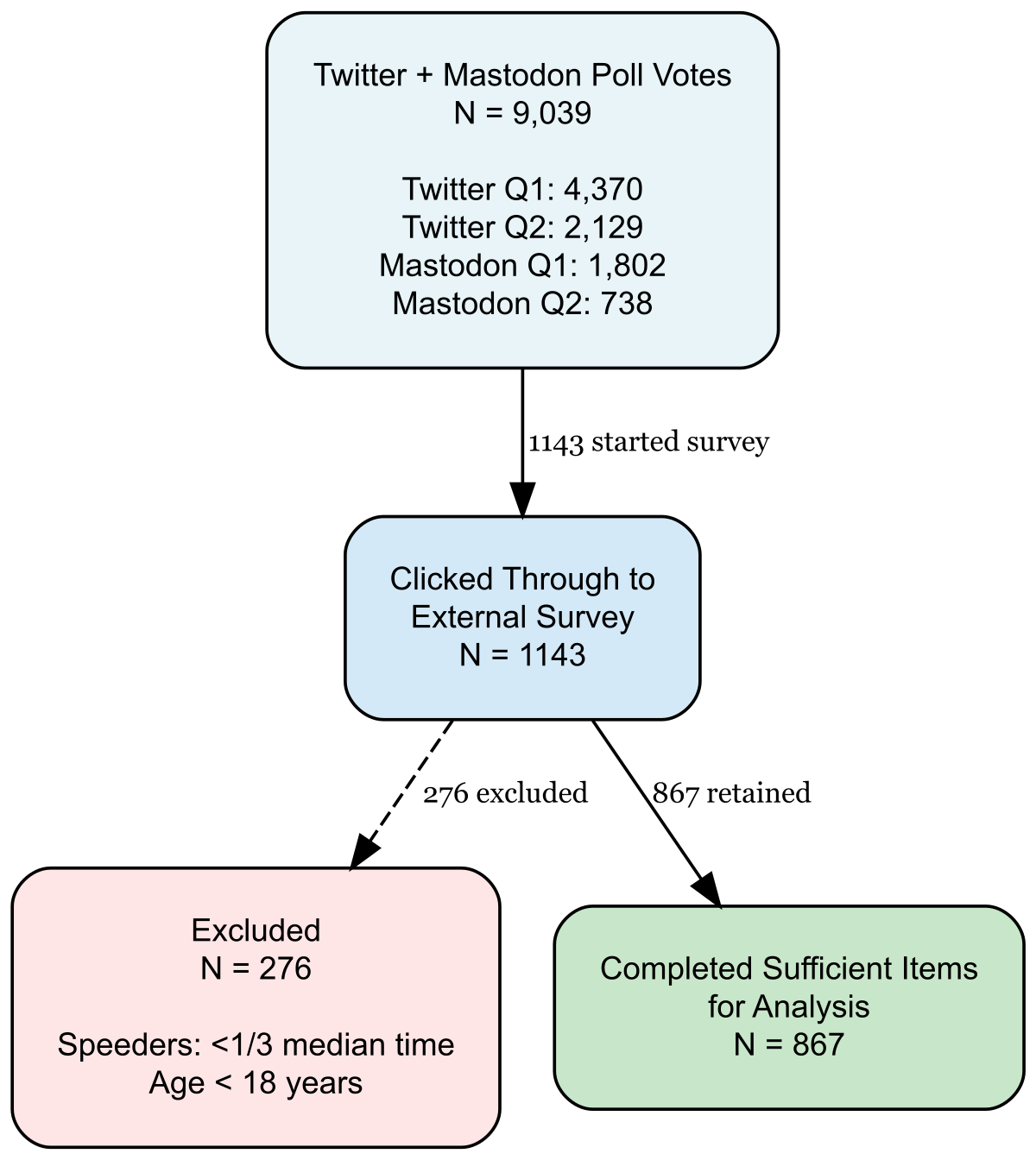


**Figure S6:** Participant flow diagram for the comparative COVID-19 study, showing the number of social media poll (SMP) voters, those who clicked through to the external survey, participants completing sufficient items for analysis, and reasons for exclusion. SMP voters represent any Twitter or Mastodon users in Germany who viewed the poll between July 19 and July 26, 2023; external survey data were collected between July 18 and August 30, 2023.

The proportion of missing data was examined for key variables. No missing values were observed for Gender or the Number of Infections. A small proportion of cases (1.5%) had missing information for Age and some for infection-related dates: Date of the First Infection (0.21%), Date of the Second Infection (1.22%), and Date of the Third Infection (11.11%). This proportion of missing data for infection dates did not impact our analyses, as these dates were only used to estimate incidence within a defined time frame. Since the analyses were based on aggregated counts rather than individual-level temporal data, missing infection dates did not bias the overall incidence estimates. Only COVID-19 Vaccination Status showed a considerable amount of missing data (34.6%), although here unvaccinated participants may be included.


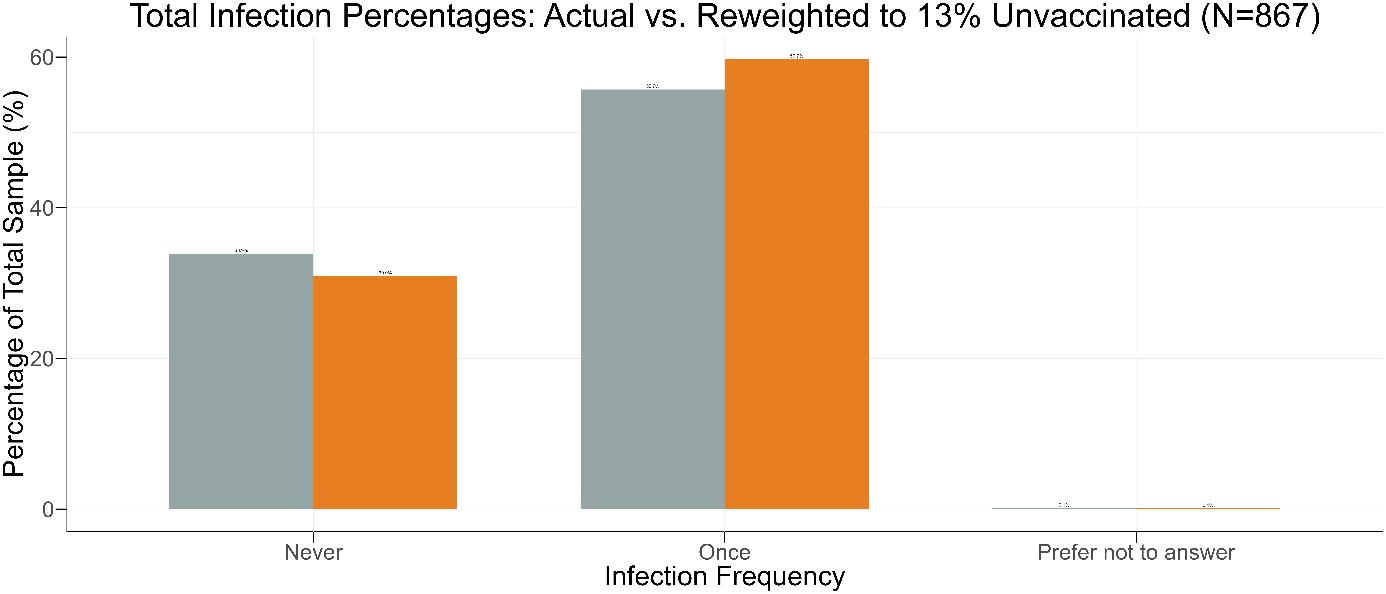


**Figure S7:** Bootstrapped comparison of COVID-19 infection frequencies between vaccinated and unvaccinated participants in the comparative study. Data originate from social media users in Germany who completed the external survey between July 18 and August 30, 2023.

Using Bootstrapping for the infection numbers to achieve a 13% unvaccinated participant rate results in only a minor change: a three-percentage-point decrease in the share of participants reporting never having been infected (from 33.9% to 30.9%) and a four-percentage-point increase in those reporting a single infection (from 55.7% to 59.7%).


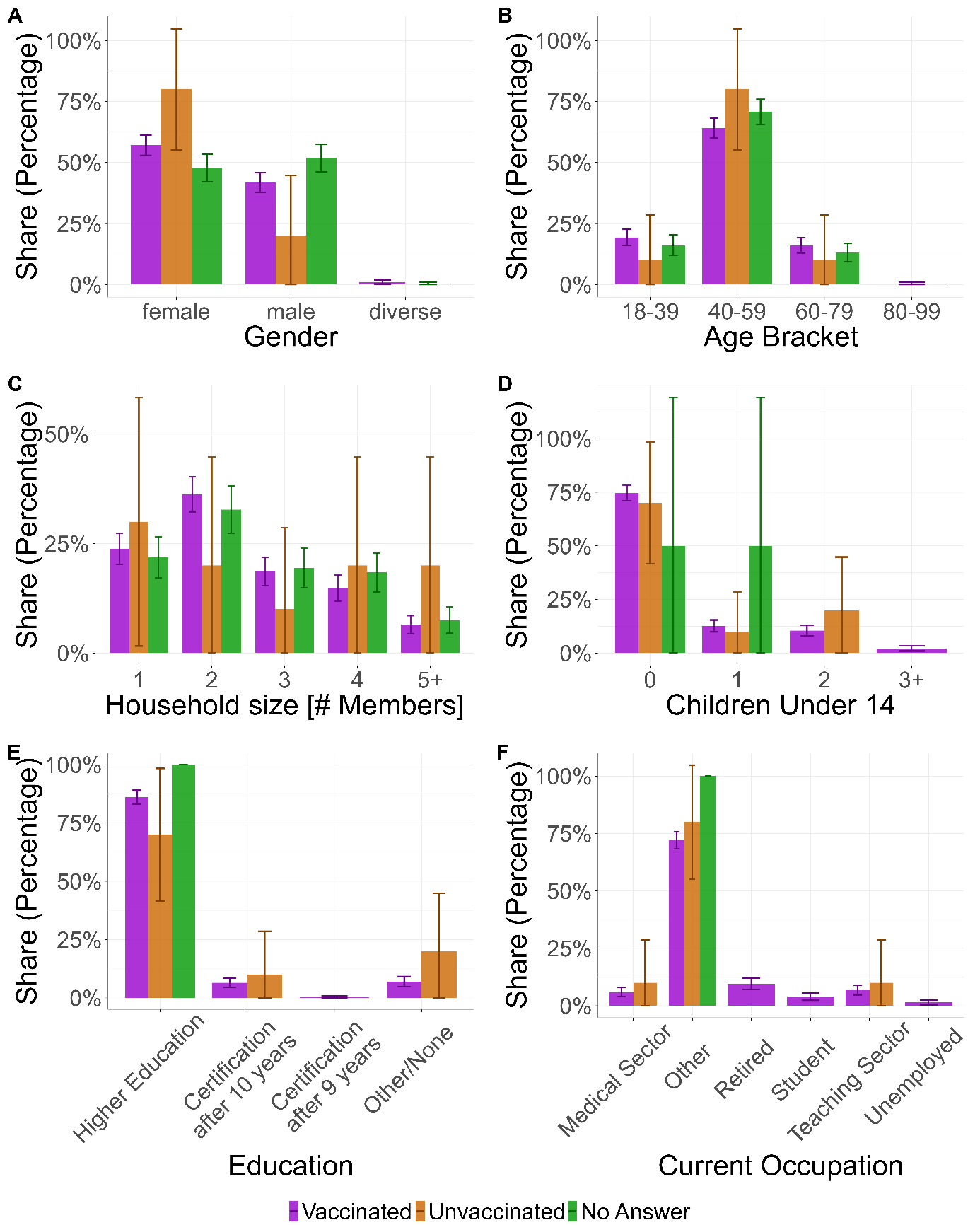


**Figure S8:** Demographic comparison of vaccinated, unvaccinated, and non-reporting participants in the comparative COVID-19 study. Data were collected from social media users in Germany who accessed the external survey between July 18 and August 30, 2023.

| Wenn ja: Wie viele Impfungen haben Sie bekommen und mit welchem Impfstoff wurde geimpft? | If yes: How many vaccinations did you receive and which vaccine was used? | Matrix mit: Erste, Zweite, Dritte, Vierte Impfung | Matrix with: First, second, third and fourth vaccination and which vaccine was used? | Wenn ja: Wie viele Impfungen haben Sie bekommen und mit welchem Impfstoff wurde geimpft? | If yes: How many vaccinations did you receive and which vaccine was used? | Matrix mit: Erste, Zweite, Dritte, Vierte Impfung | Matrix with: First, second, third and fourth vaccination and which vaccine was used? |
| --- | --- | --- | --- | --- | --- | --- | --- |
| Haben Sie bereits eine COVID-19-Impfung bekommen? | Have you already had a COVID-19 vaccination? | Ja, Nein, Weiß ich nicht | Yes, No, I don’t know | Haben Sie bereits eine COVID-19-Impfung bekommen? | Have you already had a COVID-19 vaccination? | Ja, Nein, Weiß ich nicht | Yes, No, I don’t know |
| Wann wurde die erste Infektion mit dem Coronavirus (SARS-CoV-2/ COVID-19) festgestellt? Falls Sie das genaue Datum nicht mehr wissen, geben Sie ein ungefähres Datum an. | When was the first infection with coronavirus (SARS-CoV-2/ COVID-19) detected? If you don’t know the exact date, please give an approximate date. | Pro Infektion ein Datum (Tag, Monat, Jahr) | Per infection with date (day, month, year) | Wann wurden Sie erstmals positiv auf SARS-CoV-2 getestet? | When have you tested PCR-positive (with date)? | Eingabe pro Datum seit Februar 2020 (positiv getesteter PCR-Test); wenn Sie sich nicht mehr genau erinnern, geben Sie nur den Monat an. | Input by date (day, month, year), February 2020 (if you don’t remember the day, just give the month) |
| Wie häufig waren Sie schon (positives Testergebnis) mit dem Coronavirus (SARS-CoV-2) infiziert/ COVID-19? | How often have you tested positive for coronavirus (SARS-CoV-2/ COVID-19)? | Nie, Einmal, Zweimal, Dreimal, Mehr als dreimal | Never, Once, Twice, Three times, More than three times | Wie häufig wurden Sie schon positiv auf das Coronavirus (SARS-CoV-2/ COVID-19) getestet? | How often have you tested positive for coronavirus (SARS-CoV-2/ COVID-19)? | Nie, Einmal, Zweimal, Dreimal, Mehr als dreimal | Never, Once, Twice, Three times, More than three times |
| Question External Survey [German] | Question External Survey [translated] | Answer Options External Survey [German] | Answer Options External Survey [translated] | Question MuSPAD [German] | Question MuSPAD [translated] | Answer Options MuSPAD [German] | Answer Options MuSPAD [translated] |

**Table S3:** Comparison of External Survey and MuSPAD Questions and Answers used with participants in the comparative COVID-19 study. Data were collected from social media users in Germany who accessed the external survey between July 18 and August 30, 2023.
